# Film-Forming Corrosion Inhibitor of ZnAl Layered Double Hydroxide Intercalated with Mussel Adhesive Protein

**DOI:** 10.3390/molecules30173480

**Published:** 2025-08-25

**Authors:** Yanhui Cao, Dajiang Zheng, Fan Zhang, Jinshan Pan, Changjian Lin, Jingjing Wang, Congshu Huang

**Affiliations:** 1 National Key Laboratory of Marine Corrosion and Protection, Luoyang Ship Material Research Institute, Xiamen 361101, China; yanhuicao@vip.163.com (Y.C.); jingjing811014@163.com (J.W.); hcs05@126.com (C.H.); 2 State Key Laboratory of Physical Chemistry of Solid Surfaces, College of Chemistry and Chemical Engineering, Xiamen University, Xiamen 361005, China; 3 College of Materials, Xiamen University, Xiamen 361005, China; 4 Department of Engineering and Design, School of Engineering and Informatics, University of Sussex, Brighton BN1 9RH, UK; zhangfan@sussex.ac.uk; 5 Division of Surface and Corrosion Science, Department of Chemistry, KTH Royal Institute of Technology, SE-100 44 Stockholm, Sweden; jinshanp@kth.se

**Keywords:** aluminum, layered double hydroxide, corrosion protection

## Abstract

In order to enhance the corrosion resistance of aluminum alloys, mussel adhesive protein (MAP) was intercalated into layered double hydroxide (LDH) grown onto an Al substrate. The results from X-ray diffraction (XRD), attenuated total reflectance Fourier transform infrared spectroscopy (ATR-FTIR) and energy dispersive spectroscopy (EDS) measurements all confirmed that part of the positively charged MAP can be successfully intercalated into the LDH based on the strong second reactivity. MAP is able to form complexes with the metal cations and hydroxides, leading to less positive charges on the hydroxide layers of the LDH. The intercalation results in the removal of the previously intercalated anions from the interlayer space of the LDH, which maintains the charge balance and lamellar structure. The MAP intercalated LDH film can provide effective corrosion protection to the Al substrate.

## 1. Introduction

Layered double hydroxide (LDH) has drawn increasing attention as a novel strategy for corrosion protection due to its special two-dimensional lamellar structure and capability of intercalation of various corrosion inhibitors. The lamellar structure of LDHs consists of hydroxide layers of divalent and trivalent cations. The general formula of LDH can be expressed as [M_1−x_^2+^M_x_^3+^(OH)^2^]^x+^(A^n−^)_x/n_ mH_2_O, where M^2+^ and M^3+^ are di- and trivalent cations, respectively, A^n−^ is the anion, and x is the molar ratio of M^2+^ and M^3+^ [1,2]. The cation hydroxide layers [M_1−x_^2+^M_x_^3+^(OH)_2_]^x+^ are positively charged and neutralized by the anions in the interlayer space (also called the gallery of LDHs).

The unique structure of LDHs endows itself with significant anion intercalation capacity. A wide variety of anions have been intercalated successfully in the interlayer space as reported in the literature, in both types of inorganic anions and organic anions [3,4,5,6]. The intercalated anions interact with the hydroxide layers through electrostatic attractions, hydrogen bonds and dispersive forces, all of which belong to weak interactions [1]. Therefore, the anion-exchange reaction may occur between the anions in the gallery of LDHs and anions in the surrounding environment. Parameters including the size, charge and concentration of the anions can significantly influence the equilibrium of the anion-exchange reaction. The smaller anions in the environment preferentially tend to replace the anions of a larger size in LDHs, if they have the same charges [7]. Moreover, anions with higher charge are more likely to be intercalated into the interlayer space of LDHs due to a stronger electrostatic interaction with the hydroxide layers [8]. Further, the exchanged amount of the anions increases with the increase in the anion concentration in the environment [9].

Based on the anion-exchange characteristic, LDHs loaded with inhibitory anions have been widely used for the corrosion protection of metals in coatings and concrete [2,10,11]. The inhibitory anions can be released into the environment upon the intercalation of aggressive anions such as chloride anions. The responsive inhibitory anions releasing and aggressive anions absorbing behaviors of LDHs provide excellent corrosion protection with a self-healing property to the metal structure. The self-healing property of the coating is able to increase the service life and reduce the cost and effort of repairing or reconstruction in practical application.

**Figure 1:**
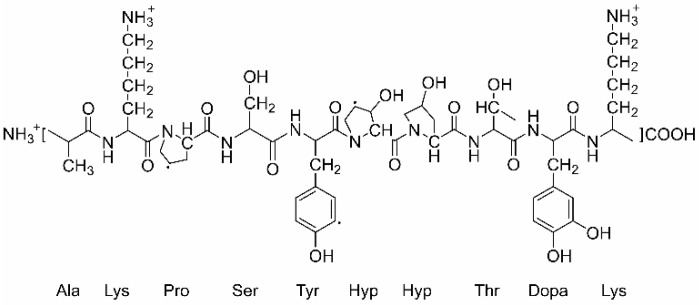
The chemical structure of the decametric repeating unit of Mefp-1 [13].

In recent years, mussel adhesive proteins (MAPs) extracted from Mytilus edulis have received much interest due to their strong adhesive property on various substrates. Mefp-1(Mytilus edulis foot protein) is the first protein extracted and identified from the Mytilus edulis foot. It is a large protein with a molecular weight of 108 kDa. The isoelectric point (pI) of Mefp-1 is 10, thus, it is positively charged in neutral and acidic environments [12]. The repetitive sequence of the Mefp-1 molecule consists of 75–85 repeating units shown in Figure 1 [13]. Dihydroxyphenylalanine (DOPA) is the primary residue of Mefp-1, and the adhesion and cohesion properties of the molecules appear through its interfacial interactions including hydrogen bonding, covalent bonding, metal-ligand, etc. Mefp-1 could form strong complexes with metal ions, e.g., Fe^3+^ could bind one, two or three catechols of DOPA. The complexation with iron ions can enhance the cohesion of the preformed Mefp-1 film, and thus lead to the compaction of the film [14].

To the best of our knowledge, however, the intercalating of inhibitors into the gallery of LDHs has only been limited within the negatively charged species, due to the positively charged nature of the hydroxide layers. The intercalation of positively charged species into the gallery of LDHs has been rarely reported. Hyoung-Jun Kim reported that a cationic anticancer drug (doxorubicin (DOX)) had been intercalated into the gallery of the LDH, where polyacrylic acid (PAA) has been intercalated into the LDH first and the use of anionic PAA could gave rise to extra negative charges following the intercalation. The electrostatic attraction between DOX and the newly produced negative charges led to the successful intercalation of DOX into the gallery [15]. Mefp-1 can adsorb on metal and metal oxides strongly, and form a film on the surface. Our previous studies show that the net positively charged Mefp-1 is even able to adsorb onto the positively charged surface and show the corrosion protective and self-healing properties to metallic materials [16], which motivate the intercalation of Mefp-1 into the LDH.

This work is inspired by the universal adhesive and corrosion protective properties of Mefp-1. We successfully intercalated part of the positively charged inhibitor Mefp-1 into the LDHs through the simple immersion procedure. A range of surface characterization and electrochemical techniques have been applied to obtain insight into the mechanisms of Mefp-1 intercalation and evaluate the corrosion inhibition property of the formed LDHs/Mefp-1 composite film. It is of obvious significance for further development of new environmentally friendly and self-healing film-forming corrosion inhibitors based on LDHs and biomass.

## 2. Results and Discussion

### 2.1. Characterizations of the LDH Films Intercalated with Mefp-1

**Figure 2:**
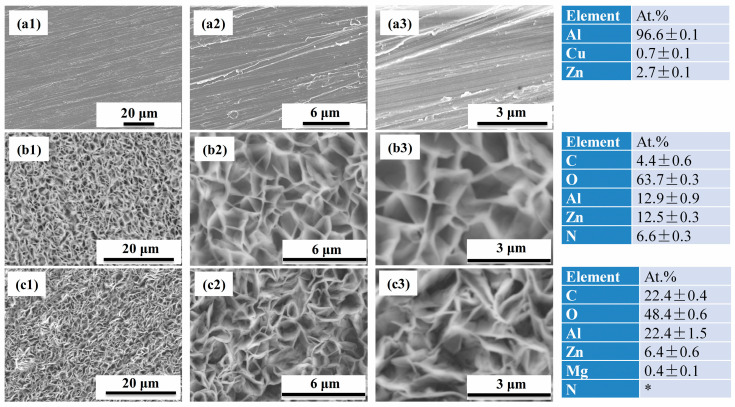
SEM and EDS results of (**a1**–**a3**) AA7075, (**b1**–**b3**) ZnAl-LDH, and (**c1**–**c3**) ZnAl-LDH-Mefp-1 (* means the content of N was below the detection limit).

Figure 2 shows the scanning electronic microscopy (SEM) morphology and Energy dispersive spectroscopy (EDS) elemental compositions of AA7075, ZnAl-LDH film, and ZnAl-LDH-Mefp-1 film. As can be seen from Figure 2(a1–a3), scratches can be found on the blank substrate caused by the sandpaper grinding and the corresponding EDS results indicated the main composition of Al, Zn, and Cu. According to Figure 2(b1–b3), the LDH platelets grew perpendicularly on the surface and presented a nest-like structure, which can be explained by the “evolution selection mechanism” [17]. After immersion in Mefp-1 solution, as shown in Figure 2(c1–c3), the LDH film maintained its perpendicular nest structure, but the platelets became more compressed. The maintaining morphology demonstrated that the treatment of ZnAl-LDH film with Mefp-1 does not give rise to any destruction of the LDH structure. Moreover, the Mefp-1 treatment induced a compaction of the platelets which suggests that the Mefp-1 entered into the interlayer of LDH, leading to a significant stress in the film due to the large molecular size [18,19].

According to the EDS result, the amount of C element in the coating increased largely after the treatment of Mefp-1, indicating the existence of Mefp-1 in the ZnAl-LDH-Mefp-1 films. In addition, for the ZnAl-LDH film, the measured N elemental content was about 6.6%, which originates from the nitrate content in the interlayer of the LDH. However, although Mefp-1 contains a certain amount of N, N cannot be detected by the EDS. The possible reasons may be that the content of N was too low or the N peak was hidden in some large peak. The significant decrease in the N content after the treatment of Mefp-1 suggests that most of the nitrate content has been removed from the interlayer space upon the Mefp-1 intercalation. In addition, the Zn/Al ratio decreased from 1.0 to 0.3 upon the treatment of Mefp-1, which can be explained by the thinning of the pre-formed LDH film during the immersion in acidic Mefp-1 solution, where more of a signal of Al from the substrate can be detected.

**Figure 3:**
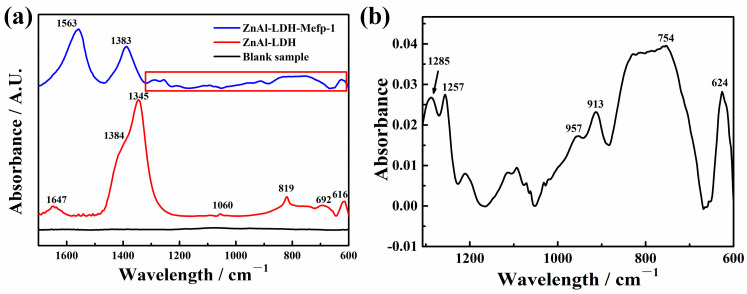
ATR-FTIR spectra of (**a**) AA7075, ZnAl-LDH, and ZnAl-LDH-Mefp-1 films, (**b**) is the magnification of the spectrum marked with the red frame in (**a**).

The Attenuated total reflectance Fourier transform infrared spectroscopy (ATR-FTIR) results of the LDH coatings before and after Mefp-1 intercalation are shown in Figure 3. The AIR-FTIR result of AA7075 is also presented for comparison (the black line in Figure 3a) and the result indicated that almost no characteristic peaks can be observed. In Figure 3a, before the Mefp-1 treatment, the peak at 616 cm^−1^ can be attributed to M-OH vibrations in the lattice of LDH [20,21]. The peaks at 692 and 819 cm^−1^ belong to the in-plane and out-plane bending of carbonates, respectively [22]. The peak at 1060 cm^−1^ can be assigned to the symmetric stretching of CO_3_^2–^ [22]. Moreover, the intensive peak at 1345 cm^−1^ and its shoulder at 1384 cm^−1^ can be ascribed to the co-intercalation of carbonates and nitrates [20]. The peak at 1647 cm^−1^ is due to the bending vibration of the interlayer water [21,23]. For the sample after Mefp-1 treatment, the peak at 1563 cm^−1^ can be ascribed to the N-H bending vibrations of amide Ⅱ and the asymmetric carboxylate stretch [14,24], which is evidence for the existence of Mefp-1 in LDH films. The peak at 1647 cm^−1^ almost disappeared after treatment by Mefp-1, which may be caused by the occurrence of dehydration and contraction of the film due to the complexing with metal ions [14]. Moreover, as shown in Figure 3a the relative intensity of the nitrates at 1383 cm^−1^ decreased significantly upon the treatment of Mefp-1, indicating a decreased amount of the nitrates in the interlayer space of the LDH [20]. Figure 3b presents the enlarged spectrum marked in Figure 3a, which provides more evidence of Mefp-1 intercalated in the LDH. All the peaks corresponding to carbonates disappeared completely due to the immersion of LDH film in the acidic Mefp-1 solution. The peak at 624 cm^−1^ can be attributed to M-OH vibrations in the layer [25]. The peak at 754 cm^−1^ was caused by the C-H out-of-plane bending vibrations of the benzene ring, indicating the presence of Mefp-1 as it contains the residues of Tyr and Dopa as shown in Figure 1 [26]. The peaks at 913 and 957 cm^−1^ can be ascribed to the DOPA residue [14]. In addition, there were two peaks at 1257 and 1285 cm^−1^, which can be attributed to the C-O stretching in the DOPA functional groups, verifying the existence of Mefp-1 on LDH [14,27,28].

The above ATR-FTIR results convincingly verified the existence of Mefp-1 in LDHs, and is in good agreement with the EDS result showing that a significant number of nitrates are removed from the interlayer space of LDH due to the intercalation of Mefp-1.

**Figure 4:**
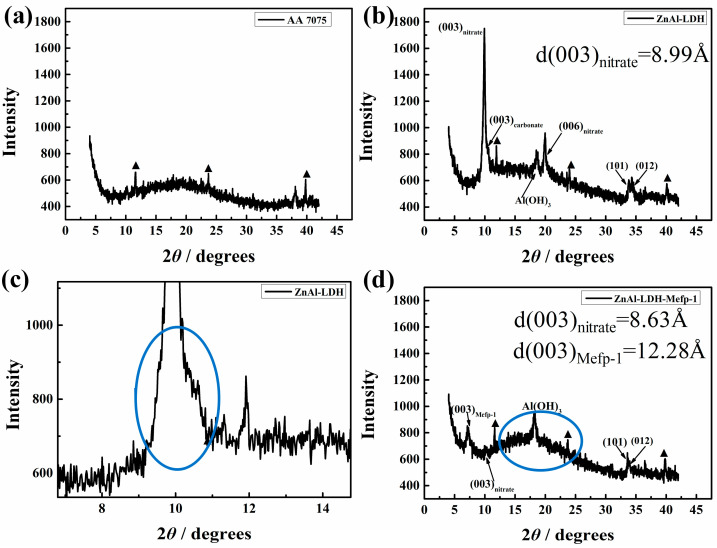
XRD spectra of (**a**) AA7075, (**b**) and (**c**) ZnAl-LDH, and (**d**) ZnAl-LDH-Mefp-1.

Figure 4 shows the X-ray diffraction (XRD) spectra of different prepared samples. The spectrum of the aluminum substrate presented three main characteristics peaks, as marked with triangles in the spectra of Figure 4a [29]. The XRD spectrum of ZnAl-LDH presented major peaks at 9.89 and 19.93°, assigned to the (003) and (006) planes of LDH intercalated with nitrates originating from Zn(NO_3_)_2_ and NH_4_NO_3_ [30]. The corresponding basal spacing was calculated to be 0.90 nm based on the Bragg’s equation, verifying that nitrates have been loaded in the gallery [17,31,32]. As the preparation was performed without inert gas protection, therefore, the small right shoulder of the asymmetrical peak marked with the blue circle in Figure 4c can be ascribed to the co-intercalated carbonates in ZnAl-LDH [33]. After the treatment with Mefp-1 solution (Figure 4d), the obtained spectrum also shows typical peaks of LDH, indicating the well-preserved structure of LDH, in good accordance with the SEM result. The peak corresponding to nitrates around 10.0^0^ decreased markedly, which may be associated with two reasons. Firstly, lots of intercalated nitrates were removed from the LDH gallery, confirmed by the above EDS and FTIR measurements. Secondly, the LDH structure experienced dissolution during the Mefp-1 treatment process, in agreement with the decreased Zn/Al ratio observed from the EDS result. The shoulder peak at 10.6° corresponding to carbonates disappeared after Mefp-1 treatment. The disappearance of the co-intercalated carbonates is most likely due to the decarbonated reaction promoted by H^+^ in the weak acidic Mefp-1 solution [34]. Consequently, the loaded carbonates were eliminated from the gallery. The disappearance of carbonates is in good accordance with the results of FTIR and EDS.

A peak at 7.19° appeared upon the immersion in the Mefp-1 solution as shown in Figure 4d, the corresponding basal spacing was expanded to 1.23 nm as calculated according to the Bragg’s equation, which was increased by 0.33 nm compared to the original value of 0.9 nm. This result suggested that intercalation of new species with larger sizes in comparison with nitrates into the LDH gallery. A broad peak at around 20° marked with a blue circle in Figure 4d can be observed on the ZnAl-LDH-Mefp-1 sample, attributed to the Dopa residues of Mefp-1, which has been reported in the literature [35]. Therefore, the XRD results further verified the existence of DOPA residues of Mefp-1 in ZnAl-LDH-Mefp-1. Mefp-1 is a large molecule with a complex sequence, as shown in Figure 1, including charged residues (Lys), and adhesive but non-charged residues (DOPA). In this study, the basal spacing increases about 0.33 nm upon the intercalation of Mefp-1, which is much smaller than the reported hydrodynamic radius RH of the Mefp-1 molecule [36]. Therefore, given the small basal spacing of the LDH and the evidence of Mefp-1 in the ZnAl-LDH-Mefp-1 film, it is reasonable to conclude that only some segments of the Mefp-1 molecule are intercalated into the LDH gallery. The intercalated segments are most likely to be DOPA residues given their adhesive nature that facilitates the formation of a strong bond with the metal cations of the hydroxide layers. As shown in Figure 4d, the low intensity of the (003) peak of nitrate suggests that plenty of the nitrates are removed from the interlayer gallery of ZnAl-LDH-Mefp-1. On one hand, the decrease in the nitrates and the disappearance of carbonates will provide extra space for the intercalation of Mefp-1. On the other hand, to maintain electric neutrality, the removal of these negatively charged species in the interlayer space should correspond to less positive charges in the hydroxide layers. All these facts provide a possibility for the interaction with the hydroxide layers and the intercalated DOPA residues of Mefp-1.

Although it is well known that only the negatively charged species can be intercalated into the gallery with positively charged hydroxides of LDHs, our study firstly provides evidence that part of the net positively charged molecules of Mefp-1 are able to intercalate into the ZnAl-LDH based on its stronger driving force of chemical reaction. The positive charges of the metal cations decrease upon complexation with Mefp-1 molecules. Thus, the number of positive charges on the hydroxide layers of ZnAl-LDH-Mefp-1 would be smaller compared with that of LDH without the modification of Mefp-1. Accordingly, the amount of negative charges in the interlayer space should become smaller to maintain the charge balance. All the XRD, FTIR, and EDS results discussed above demonstrate that plenty of nitrate anions can be removed from the interlayer space, which not only made extra space for the intercalation of Mefp-1 but also played a significant role in realizing the charge balance of the whole system.

### 2.2. Corrosion Protective Performance of ZnAl-LDH-Mefp-1 Film on Al Substrate

In order to investigate the corrosion protection ability of Mefp-1 intercalated LDH film, Electrochemical Impedance Spectroscopy (EIS) measurements have been performed in 3.5 wt.% NaCl solution.

**Figure 5:**
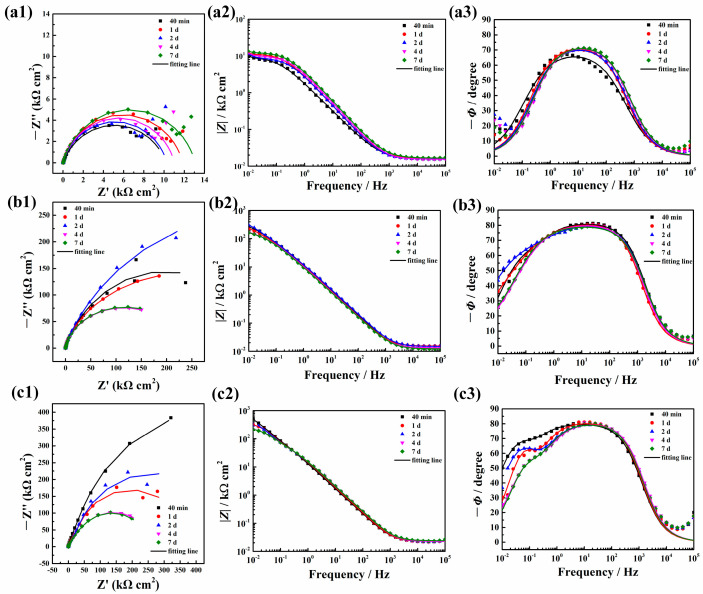
EIS spectra of blank Al substrate (**a1**–**a3**), Al substrate with pre-formed ZnAl-LDH (**b1**–**b3**), and Al substrate with pre-formed ZnAl-LDH-Mefp-1 (**c1**–**c3**) after different immersion time in 3.5 wt.% NaCl solution.

The corresponding EIS spectra including Nyquist plots and Bode plots are shown in Figure 5. As can be seen from the Nyquist plots in Figure 5 (a1–c1), the blank Al substrates presented a capacitive arc with a small tail corresponding to the Warburg resistance while both of the other two samples exhibited only one capacitive arc. The capacitive arc of ZnAl-LDH-Mefp-1 was the largest followed by ZnAl-LDH, and the arc of the blank substrate was the smallest. According to Figure 5(a2–c2), the impedance modulus at a low frequency of the ZnAl-LDH-Mefp-1 was one magnitude higher than that of the Al substrate, indicating effective protection of the film towards the substrate. In addition, the impedance was larger to some extent in comparison with the ZnAl-LDH film.

**Figure 6:**
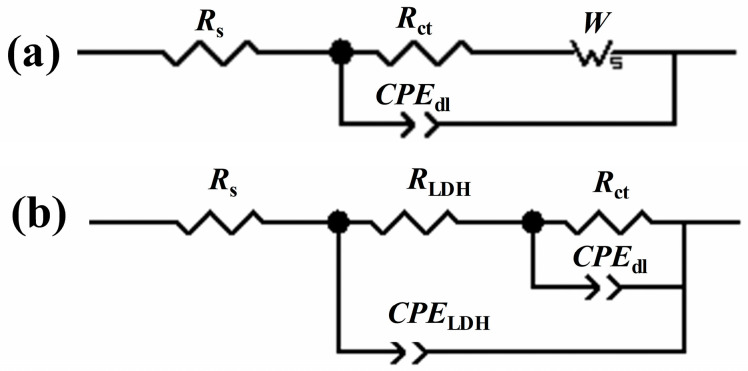
Equivalent circuit used to simulate the EIS data (**a**) for blank Al substrate, (**b**) for ZnAl-LDH and ZnAl-LDH-Mefp-1.

According to Figure 5(a3–c3), all the frequency-phase angle curves present two phase angle peaks. For the blank sample, the peak in the frequency range of 10^3^–10^−1^ Hz was ascribed to the charge transfer process occurring in the solution/substrate interface. The peak at 10^−2^ Hz can be attributed to the mass transport controlled processes due to the accumulation of corrosion product [37,38]. Notably, this peak was incomplete and poorly defined, which was due to the fact that the frequency of 10^−2^ Hz was not low enough. This observation was in good accordance with the phenomenon reported in the literature [37,38]. In Figure 5(b3,c3), the peak in the middle frequency from 10^0^–10^3^ Hz can be attributed to the LDH layer on the substrate surface and the peak in the low frequency (10^−2^–10^0^ Hz) was ascribed to the electrochemical corrosion process occurring on the LDH layer/substrate interface [39,40,41]. Therefore, the equivalent circuits in Figure 6 with two time constants were adopted to simulate the EIS data. In Figure 6a, *R*_s_ is the solution resistance, *R*_ct_ is the charge transfer resistance related to the electrochemical corrosion process, and *CPE*_dl_ is used instead of an ideal capacitance to represent the double layer capacitance. W is the Warburg resistance related to the mass-controlled process. In Figure 6b, *R*_LDH_ is the resistance of the LDH layer, and *CPE*_LDH_ is the capacitance of the LDH layer. Similarly, *R*_ct_ is the charge transfer resistance, and *CPE*_dl_ is related to the double layer capacitance.

The fitting results are listed in Table 1, Table 2 and Table 3. As can be seen from Table 1, the values of *R*_ct_ decreased slightly after immersion for 1–4 d in comparison with that after immersion for 40 min. *R*_ct_ increased slightly after immersion for 7 d, which can be ascribed to the accumulation of corrosion products on the surface. The values of W increased gradually along with the increase in immersion time to 2 d and decreased slightly after immersion for 4 and 7 d. The element of *CPE* was represented by *Υ*_0_ and n. It can be seen that *Υ*_0_ decreased slowly upon the immersion time, while n increased after immersion for 1 d and then remained almost unchanged afterwards. This result was probably due to the fact that the produced corrosion product could act as a protective film at the electrolyte/substrate interface and decrease the heterogeneity of the surface to some extent [42,43]. According to Table 2 and Table 3, both the *R*_LDH_ and *R*_ct_ values of ZnAl-LDH-Mefp-1 were higher than those of the ZnAl-LDH samples. In addition, the *R*_s_ values were also slightly higher in comparison to those without modification by Mefp-1, indicating that the LDH layer became denser and it became more difficult for the water molecules to enter into the LDH layer. In addition, the *Y*_0_ values of both the LDH layer capacitance and the double layer capacitance of ZnAl-LDH-Mefp-1 were significantly smaller than those of ZnAl-LDH, while the *n* values of both the LDH layer capacitance and the double layer capacitance of ZnAl-LDH-Mefp-1 were obviously larger than those of ZnAl-LDH, suggesting a more compact and denser surface in the case of ZnAl-LDH-Mefp-1 [42,43]. The above result demonstrated that the intercalation of Mefp-1 into the gallery of LDH was able to lead to the formation of a more compact LDH film against the attack of water molecules and aggressive chlorides in the environment.

Polarization resistance, *R*_p_, can be used as an indicator to characterize the corrosion resistance of the substrates in NaCl solution [44,45,46]. In the case of the blank substrate, *R*_p0_ is the sum of the charge transfer resistance (*R*_ct_) and *W* (Warburg resistance) [47,48]. For the case of the ZnAl-LDH and ZnAl-LDH-Mefp-1, *R*_p_ is the sum of the LDH layer resistance and the charge transfer resistance (*R*_ct_). The related inhibition efficiency (η(%)) is calculated according to the following equation [44,45,46,47,48]:

(1) $$\eta \left(\%\right)=\frac{R_{P}-R_{P0}}{R_{P}}\times 100\%$$

According to the above equation, the values of *η*(%) of ZnAl-LDH and ZnAl-LDH-Mefp-1 are calculated to be 86.4%, 85.8%, 80.7%, 65.0%, 70.5% and 94.0%,88.5%,80.9%,79.4%, and 82.7% at the immersion of 40 min, 1 d, 2 d, 4 d, and 7 d, respectively, indicating a relatively higher inhibition efficiency of the LDH films with Mefp-1 modification.

**Figure 7:**
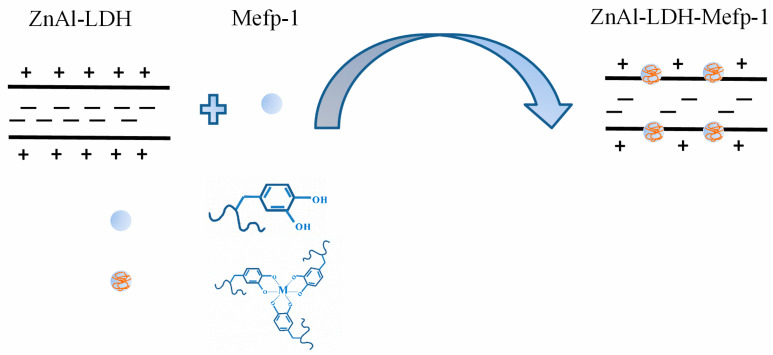
The schematic illustration of the interaction mechanism between ZnAl-LDH and Mefp-1.

The above result demonstrated the effective enhancement of Mefp-1 on the corrosion inhibition property of ZnAl-LDH film, which can be attributed to the interaction between the ZnAl-LDH film and Mefp-1 as shown in Figure 7. According to Figure 7, the catechol in the Mefp-1 underwent a complexing reaction with metal ions in the hydroxide layers and substrate-released metal ions [16], thus leading to an enhanced corrosion protection property due to the formation of a more compact physical barrier.

## 3. Materials and Methods

### 3.1. Materials

The AA7075 samples with a chemical composition (in wt.%) of 0.17% Si, 0.19% Fe, 1.6% Cu, 0.04% Mn, 2.3% Mg, 0.18% Cr, 0.01% Ni, 5.5% Zn, 0.02% Ti, 0.01% Pb, and Al (bal.) were used as the substrate. The specimen was supplied by Sapa Profiles, Sweden. The sample surface was ground with SiC grinding paper from 360, 580, 800 to 1200-grit size successively. After grinding, the AA7075 samples were ultrasonically cleaned in ethanol and then dried in air.

The chemical agent of NH_4_NO_3_ was purchased from KEBO Lab, NaCl, 25% NH_3_·H_2_O and citric acid were from Sigma-Aldrich Company, and Zn(NO_3_)_2_ was from Alfa Aesar (Johnson Matthey Company). Mefp-1 in 1 wt.% citric acid solution with an original concentration of 2 mg/mL were purchased from Biopolymer AB of Sweden. All the solutions were prepared with deionized water.

### 3.2. Preparation of LDH Films on AA7075 Substrate

A mixed solution with Zn(NO_3_)_2_ and NH_4_NO_3_ was prepared, whose concentration was 0.05 M and 0.3 M, respectively. The pH was adjusted to 6.5 through the dropwise addition of 1 wt.% NH_3_·H_2_O. The Al substrates were perpendicularly immersed into the mixture solution for 8 h at 85 °C in a Muffle furnace. After that, the samples were rinsed with deionized water and ethanol successively, and denoted as ZnAl-LDH.

For LDH intercalation, the Mefp-1 solution was diluted with 1 wt.% citric acid to the concentration of 0.1 mg/mL, and pH was adjusted with NaOH to 6.5. The Al samples with pre-formed ZnAl-LDH film were immersed in the 0.1 mg/mL Mefp-1 solution for 40 min at room temperature. Then they were gently rinsed with deionized water and dried in air naturally, and denoted as ZnAl-LDH-Mefp-1.

### 3.3. Characterizations of LDH Films

X-ray diffraction (XRD, PANalytical X’Pert PRO, Netherland PANalytical Company, Alemlo, Netherland) patterns of the LDH samples were collected with an irradiation source of Cu Ka (λ = 1.54 Å). The operation voltage was 40 kV, and the current was 40 mA. The scan range was from 3° to 80° with a scan rate of 0.04 °/s. The XRD patterns at the range of 3–42° were plotted to highlight the characteristic identification peaks of the LDHs.

The Mefp-1 intercalated in LDHs were analyzed by attenuated total reflectance Fourier transform infrared spectroscopy (ATR-FTIR) measurements using a LUMOS FTIR (Bruker Optik GmbH, Bruker Corporation, Karlsruhe, Germany) spectrometer with a nitrogen-cooled MCT detector and a diamond crystal. A total of 64 scans over wavenumber 4000–600 cm^−1^ were conducted with a resolution of 4 cm^−1^. The obtained spectra were processed and analyzed using the software of OPUS 7.5 (Bruker Optik GmbH 2014, Bruker Corporation, Karlsruhe, Germany).

The morphology and elemental composition of the LDH films were characterized by using a scanning electron microscope (SEM, FET Nova 200, America FEI company, Hillsboro, USA) coupled with an energy dispersive spectrometer (EDS, America FEI company, Hillsboro, USA). The operation voltage was 10 kV.

### 3.4. Electrochemical Impedance Spectra (EIS) Measurements

EIS measurements were carried out using an Autolab electrochemical workstation (Metrohm Limited, Herault, Switzerland) with a three-electrode system, where a KCl saturated Ag/AgCl electrode was the reference electrode and a platinum mesh was the counter electrode. The measurements were performed at open circuit potential (OCP) with a perturbation amplitude of 10 mV over a frequency range of 10^5^–10^−2^ Hz. The EIS spectra were recorded at different immersion times in 3.5 wt.% NaCl solution.

## 4. Conclusions

In this work, part of a net positively charged protein molecule was intercalated into the ZnAl-LDH gallery for the first time. The interaction mechanism was investigated by SEM/EDS, XRD, and ATR-FTIR. The SEM result suggests that the LDH structure can be maintained well after the intercalation of Mefp-1, leading to a more compact structure of the LDH. The XRD result verifies the intercalation of Mefp-1 and the release of nitrates in the interlayer space. Mefp-1 interacts with the LDH layers via the formation of complexes between catechols and the metal cations in the hydroxide layers, leading to the formation of a more compact LDH layer which could act as a better physical barrier against the attack of aggressive chlorides. In addition, the catechols of Mefp-1 were also able to react with the released metal ions during the corrosion process, resulting in the self-healing property of the system. The modification of Mefp-1 can provide an increased corrosion protection for aluminum substrate than that of the LDH film before the modification.

## Figures and Tables

**Table 1:** The fitted parameters of EIS spectra of blank Al substrate in Figure 5.

Al	*R*_s_ (Ω cm^2^)	*R*_ct_ (kΩ cm^2^)	*CPE*_dl_		*W* (kΩ cm^2^)
			*Y*_0_ (10^−5^ Ω^−1^ cm^−2^ s^n^)	*n*	
40 min	16.3 ± 0.6	11.6 ± 1.8	12.4 ± 0.4	0.78	27.0 ± 5.5
1 d	16.7 ± 0.2	10.0 ± 1.1	8.1 ± 1.4	0.83	32.7 ± 18.0
2 d	15.3 ± 0.2	7.9 ± 0.9	7.8 ± 1.2	0.84	66.3 ± 29.7
4 d	15.7 ± 0.2	9.1 ± 0.8	6.5 ± 1.1	0.83	58.5 ± 28.6
`7 d	16.6 ± 0.2	11.2 ± 0.9	5.7 ± 0.8	0.83	48.4 ± 25.0

**Table 2:** The fitted parameters of EIS spectra of Al substrate with ZnAl-LDH films in Figure 5.

ZnAl-LDH	*R*_s_ (Ω cm^2^)	*R*_LDH_ (kΩ cm^2^)	*CPE*_LDH_		*R*_ct_ (kΩ cm^2^)	*CPE*_dl_	
			*Y*_0_ (10^−5^ Ω^−1^ cm^−2^ s^n^)	*n*		*Y*_0_ (10^−5^ Ω^−1^ cm^−2^ sn)	*n*
40 min	14.0 ± 0.8	71.2 ± 9.8	1.4 ± 0.3	0.91	212.7 ± 63.6	4.1 ± 3.4	0.66
1 d	14.3 ± 0.7	41.0 ± 14.1	1.7 ± 0.4	0.90	260.4 ± 182.3	11.2 ± 12.3	0.71
2 d	13.2 ± 2.1	40.4 ± 13.1	1.5 ± 0.2	0.90	343.5 ± 292.1	1.9 ± 1.1	0.65
4 d	15.5 ± 2.3	24.0 ± 6.5	2.1 ± 0.8	0.88	168.9 ± 60.3	7.4 ± 6.1	0.63
7 d	14.7 ± 1.6	45.8 ± 19.4	2.4 ± 1.0	0.86	156.1 ± 95.7	16.3 ± 7.0	0.63

**Table 3:** The fitted parameters of EIS spectra of Al substrate with ZnAl-LDH-Mefp-1 films in Figure 5.

ZnAl-LDH-Mefp-1	*R*_s_ (Ω cm^2^)	*R*_LDH_ (kΩ cm^2^)	*CPE*_LDH_		*R*_ct_ (kΩ cm^2^)	*CPE*_dl_	
			*Y*_0_ (10^−5^ Ω^−1^ cm^−2^ s^n^)	*n*		*Y*_0_ (10^−5^ Ω^−1^ cm^−2^ sn)	*n*
40 min	19.2 ± 4.0	94.4 ± 73.0	1.2 ± 0.2	0.91	550.9 ± 276.1	1.0 ± 0.5	0.87
1 d	19.1 ± 2.8	64.7 ± 23.7	1.3 ± 0.3	0.90	305.1 ± 103.1	1.0 ± 0.4	0.89
2 d	18.9 ± 3.3	67.2 ± 26.5	1.4 ± 0.3	0.90	321.6 ± 149.7	1.4 ± 0.4	0.89
4 d	19.0 ± 1.6	40.1 ± 25.2	1.3 ± 0.1	0.92	288.1 ± 90.3	1.4 ± 0.4	0.81
7 d	19.3 ± 2.5	47.0 ± 24.3	1.4 ± 0.2	0.90	298.3 ± 125.9	1.4 ± 0.4	0.85

## Data Availability

The raw/processed data required to reproduce these findings will be shared by the corresponding author upon reasonable request.

## Abbreviations

MAPs	Mussel adhesive proteins
LDH	Layered double hydroxide
Mefp-1	Mytilus edulis foot protein
pI	Isoelectric point
DOPA	Dihydroxyphenylalanine
DOX	Doxorubicin
PAA	Polyacrylic acid
SEM	Scanning electronic microscopy
XRD	X-ray diffraction
ATR-FTIR	Attenuated total reflectance Fourier transform infrared spectroscopy
EDS	Energy dispersive spectroscopy
EIS	Electrochemical Impedance Spectroscopy
